# Appreciation and job control predict depressive symptoms: results from the Study on Mental Health at Work

**DOI:** 10.1007/s00420-021-01735-6

**Published:** 2021-06-23

**Authors:** Anne Pohrt, Daniel Fodor, Hermann Burr, Friederike Kendel

**Affiliations:** 1 Institute of Biometry and Clinical Epidemiology, Charité – Universitätsmedizin Berlin, corporate member of Freie Universität Berlin, Humboldt-Universität zu Berlin, and Berlin Institute of Health, Berlin, Germany; 2 Institute of Medical Psychology, Charité – Universitätsmedizin Berlin, corporate member of Freie Universität Berlin, Humboldt-Universität zu Berlin, and Berlin Institute of Health, Berlin, Germany; 3 Gender in Medicine, Charité – Universitätsmedizin Berlin, corporate member of Freie Universität Berlin, Humboldt-Universität zu Berlin, and Berlin Institute of Health, Berlin, Germany; 4 Federal Institute for Occupational Safety and Health (BAuA), Berlin, Germany

**Keywords:** Depressive symptom, PHQ-9, S-MGA study, Working conditions, Job control, Critical life events, Caregivers, Appreciation

## Abstract

**Objective:**

Depressive symptoms are a leading cause of disability retirement and sick leave. The aim of this study was to assess the risk of depressive symptoms in German employees and its associations with factors from both the occupational and the non-occupational domain and gender.

**Methods:**

In the second wave of the German Study of Mental Health at Work (SMGA), a representative sample of 2640 German employees (52% women) was studied. Depressive symptoms were assessed with the PHQ-9 questionnaire. Psychosocial occupational and non-occupational conditions were assessed with quantitative interviews. In this cross-sectional sample, the association of these factors with depressive symptoms was examined using logistic regression models.

**Results:**

Factors from both the occupational and the non-occupational domain were associated with risk of depressive symptoms. Low appreciation from superior (OR_men_ 2.1 (95% CI 1.2–3.7); OR_women_ 3.2 (95% CI 2.1–4.8)), low job control (OR_men_ 2.9 (95% CI 1.6–5.4); OR_women_ 1.6 (95% CI 1.0–2.5)), and critical life events (OR_men_ 3.0 (95% CI 1.6–5.4); OR_women_ 2.3 (95% CI 1.5–3.7)) had the strongest association with risk of depressive symptoms. The association with quantitative demands was stronger in caregivers than in non-caregivers. The results indicated possible differences in the associations of working conditions between men and women, and between family caregivers and non-caregivers.

**Conclusion:**

Factors from both work and private life are associated with depressive symptoms, especially appreciation, job control, and critical life events. Gender differences, with respect to appreciation and influence at work, suggest a more gender sensitive approach to psychosocial occupational health research and interventions.

## Introduction

This study examined the association of factors from both the occupational and non-occupational domain with depressive symptoms and gender. Depression is one of the major disease burdens in Germany and in the world. Next to coronary heart disease, it is the second most frequent cause of disability retirement and sick leave (Wittchen et al. 2011; Federal Chamber of Psychotherapists in Germany (BPtK) 2013; World Health Organization 2018). Depression, characterized by depressed mood, sleep disturbances and loss of energy, is, therefore, a large-scale economic issue. Prevention of depression is gaining more relevance for global health protection as well as employee health protection (Pech et al. 2010).

Risk factors for depression include bereavement, lack of social support and stress. Over recent years, many studies have focused on the effect of adverse working conditions on mental health (Stansfeld and Candy 2006; Kleppa et al. 2008; Theorell et al. 2015; Madsen et al. 2017), finding evidence that psychosocial working conditions affect the risk of developing depressive symptoms. Two models are often employed to explain the effect of working conditions on mental health: the JDC model (Job Demand Control model) which describes the effects of high demands and low control, and less often the ERI model (Effort-Reward-Imbalance model) (Siegrist 2016), which focuses on an imbalance of effort and reward. Other work factors such as appreciation by superior are considered less often (Theorell et al. 2015; Burr and d’Errico 2018). There is also evidence of the impact of non-occupational stressors such as family care obligations and critical life events (CLE) (Kessler 1997; Pinquart and Sörensen 2003; Dickau 2015), which provide evidence that family caregivers have higher risk of depression and stress and that CLE are a strong predictor of depression. (Siegrist 2016).

The prevalence of mental health problems differs between men and women (Müters et al. 2013). Depression, which affects twice the number of women as men, is a striking example. This difference has been attributed to diagnostic bias, biological patterns, and women being more likely to ruminate and internalize symptoms (Kuehner 2017). It has also been attributed to stressors that differ by gender in both occupational and non-occupational domains (Müters et al. 2013). Theorell et al.’s review (2015) examined gender effects of adverse working conditions (decision latitude, job strain, bullying) on depressive symptoms, but did not find any differences between genders. Similarly, a large German study examining the effects of ERI on risk of depression found no gender differences (Wege et al. 2018).

However, there have been very few studies examining the interplay of both occupational and non-occupational domains and gender with the risk of depression. The only recent study to our knowledge is the Dutch NEMESIS study which reported effects of both work and family roles on risk of depressive and anxiety disorders (Plaisier et al. 2008). The study found the work role to be generally protective for men’s, though not for women’s mental health. Given the lack of studies in this field, we sought to investigate the association between occupational stress factors with depressive symptoms and how these associations differ by non-occupational factors and by gender.

## Methods

### The German Study of Mental Health at Work

The German Study of Mental Health at Work (SMGA) is a representative survey study of the German working population, first conducted in 2011/2012 (Rose et al. 2017). The participants were randomly selected from social insurance data, therefore, the sample is representative for socio-demographic characteristics and includes mostly actively working employees. The response rate at baseline was 33%, with 4.511 interviews resulting from 13.590 sampled addresses. The second wave (SMGA-2) was conducted in 2017 and included 2640 participants (Lange et al. 2019). The questionnaire was revised for the second wave to include questions on family care obligations, which were then included in analysis.

### Variables

A computer-assisted personal interview collected information on gender, age, occupational and non-occupational variables. Participants answered items on depressive symptoms using a paper–pencil questionnaire in the absence of the interviewer (Rose et al. 2017). The questions are shown in Table 1.

**Table 1:** Dependent and independent variables

Domain	Variables	Questions
Occupational burdens	Full or part time work, overtime	short working hours (10 to 35 h per week), standard working hours (35 to 40 h per week), long working hours (above 40 h per week), not working (< 10 h per week)
	Appreciation by superior	To what extent… … is your work recognized and appreciated by your superior? … are you being respected by your superior? … are you being treated fairly at your workplace?
	Job control^a^	Do you have a large degree of influence on the decisions concerning your work? Do you have a say in choosing who you work with? Can you influence the amount of work assigned to you? Do you have any influence on what you do at work? Can you decide when to take a break? Can you take holidays more or less when you wish? Can you leave your work to have a chat with a colleague? If you have some private business is it possible for you to leave your work for half an hour without special permission?
	Quantitative demands	Do you have to work very fast? Is your workload unevenly distributed so it piles up? How often do you not have time to complete all your work tasks? Do you get behind with your work? Do you have enough time for your work tasks? Do you have to do overtime?
Non-occupational burdens	Family care obligations	Are you nursing someone in your own home? Are you nursing someone outside your own home?
	Living with partner	Do you live with your partner?
	Children in the household	Number of children under 14 years of age
	Critical life events	Did you have to experience one or more out of the following events in the near past: job change, removal, divorce or separation from partner, severe disease of a loved one, death of partner, death of another related person, other event
Outcome: depressive symptoms	PHQ-9	Over the past 2 weeks, how often have you been bothered by any of the following problems? Little interest or pleasure in doing things; feeling down, depressed, or hopeless; trouble falling or staying asleep, or sleeping too much; feeling tired or having little energy; poor appetite or overeating; feeling bad about yourself—or that you are a failure or have let yourself or your family down; trouble concentrating on things, such as reading the newspaper or watching television; moving or speaking so slowly that other people could have noticed? Or the opposite—being so fidgety or restless that you have been moving around a lot more than usual; thoughts that you would be better off dead or of hurting yourself in some way

^a^Composed of the COPSOQ dimensions, “Influence at work” (i.e. “decision authority”) and “Control over working time” (i.e. “degrees of freedom”)

The Patient Health Questionnaire (PHQ-9) was used to assess the severity of *depressive symptoms* (Kroenke and Spitzer 2002). This screening instrument comprises nine questions with the response options “Not at all” (0), “Several days” (1), “More than half the days” (3), and “Nearly every day” (3), which results in a sum score ranging from 0 to 27. In our sample, the PHQ-9 had a Cronbach’s alpha of 0.83, indicating good reliability. We evaluated the PHQ-9 as a dichotomous variable, using the standard cut-off point of ≥ 10. This cut-off has been shown to have better diagnostic performance for depressive disorder compared to the DSM-IV based algorithm score (Manea et al. 2015). The questionnaire was answered by 2459 persons (93% of the sample). Missing data were imputed only for depressive symptoms, where missing values in the PHQ-9 sum score were imputed based on age, gender, social class, and two variables on mood and energy from the SF-12 Health Survey; For other variables, we applied pairwise deletion. Occupational burdens were assessed as the current number of work hours per week plus several psychosocial working conditions variables. We grouped work hours into the following three categories: short (10 to less than 35 h per week), standard (35 to 40 h per week), and long (above 40 h per week). The working conditions were assessed using questions from the German version of the Copenhagen Psychosocial Questionnaire (COPSOQ) questionnaire (Nübling 2005; Kristensen et al. 2005; Burr et al. 2019). *Job control* was assessed with eight questions regarding the degree of influence on work and on working time. Each of the questions was answered on a Five-Point-Likert scale, where 1 was “always” and 5 was “never”. As the job control variable had been composed of the two COPSOQ dimensions “Influence at work” (i.e. “decision authority”) and “control over working time” (i.e. “degrees of freedom”), the scale was computed as mean of the answers, if at least two answers in each of the two dimensions were present.

*Quantitative demands* questions had the same Likert scale, at least three out of the six questions had to be answered, then the scale would again be computed as the mean value (with the fifth item reversed).

*Appreciation by superior* was measured using three questions. At least two these had to be answered, each on a Five-Point-Likert scale, where 1 was “To a very high extent” and 5 was “to a very low extent”, then we computed the scale as the mean value.

In this sample, job control had a Cronbach’s alpha of 0.77, appreciation by superior had an alpha of 0.85, and the quantitative demands scale had an alpha of 0.82. The scales for all working conditions were dichotomized at the median. For job control, the lower-half values were assigned the “High Job Control” label, while the higher-half values were assigned the “Low Job Control” label. For Appreciation by superior, the lower-half values were assigned the “High appreciation” label, while the higher-half values were assigned the “Low appreciation” label.

The non-occupational domain was represented by the following variables: *critical life events, family care obligations, living with a partner* (Yes or No), and *living with children* (Rose et al. 2017).

*CLE* were assessed as experiencing one or more of the following events in the recent past: job change, removal, divorce or separation from partner, severe disease of a loved one, death of partner, death of another related person, other event (open question). Life events were coded as a binary yes/no variable (Rose et al. 2017). *Family care obligations* were assessed dichotomously using the following two questions: “Are you nursing someone in your own home?” and “Are you nursing someone outside your own home?” If a person answered “Yes” to at least one of these questions, this person was coded as having family care obligations. One question addressed the number of children under 14 years of age and was coded into binary yes/no answers.

### Statistical analysis

We computed descriptive analyses to characterize the distributions of depressive symptoms as well as occupational and non-occupational factors, and to show the prevalence of depressive symptoms together with 95% confidence intervals in different subgroups as bar charts. For the occupational burden scales, we computed Spearman correlations with the PHQ-9 score.

Logistic regression analyses were then computed to analyze the associations of variables with risk of depressive symptoms. For both CLE and family care obligations, we computed models including interactions with occupational variables and report interaction p-values. We then stratified by variables of the non-occupational domain, CLE and family care obligations, to analyze differential effects. Models were adjusted for gender, age group, and main effects of occupational and other non-occupational burdens. Effect sizes are presented as OR (odds ratios) with 95% confidence intervals, marking coefficients with *p* ≤ *α* = 0.05 as significant. Also, we computed models including interactions of gender with occupational and non-occupational variables and report interaction *p *values. To examine possible gender differential effects, we computed gender-stratified logistic regressions, adjusted for age group and report odds ratios with 95% confidence intervals. One sensitivity analysis was conducted using the unimputed vs. the imputed PHQ-9 sum score, and another one using the continuous vs. the dichotomized occupational burden variables. SPSS 24 was used for all computations (IBM Corp. 2016).

## Results

The study sample consisted of 2640 persons. The majority of men and women were between 40 and 60 years of age, living with a partner and working. As the study had targeted employees 6 years before (sampling end December 2010), 348 participants were retired at the time of the second wave interview (mid 2017), 430 were not working 10 h or more per week. Sample characteristics are presented in Table 2.

**Table 2:** Sample characteristics

	Men (*n* = 1280)	Women (*n* = 1360)	Total (*n* = 2640)
Age group			
≤ 40 years	127 (10%)	130 (10%)	257 (10%)
41–60 years	936 (73%)	1032 (76%)	1968 (74%)
> 60 years	217 (17%)	198 (15%)	415 (16%)
Family care obligations			
Yes	117 (9%)	185 (14%)	302 (11%)
No	1161 (91%)	1172 (86%)	2333 (89) Missing: *n* = 5
Work hours			
Full time (35–40 h)	442 (36%)	322 (25%)	764 (30%)
Part time (10–35 h)	53 (4%)	519 (39%)	572 (23%)
Overtime (> 40 h)	532 (44%)	236 (18%)	768 (30%)
Not working (< 10 h)	193 (16%)	237 (18%)	430 (17%) Missing: *n* = 106
Job control			
Low	684 (62%)	518 (44%)	1202 (52%)
High	420 (38%)	668 (56%)	1088 (48%) Missing: *n* = 350
Appreciation by superior			
Low	304 (28%)	334 (29%)	638 (29%)
High	757 (71%)	794 (69%)	1551 (70%)
No superior	12 (1%)	16 (1%)	28 (1%) Missing: *n* = 423
Quantitative demands			
Low	516 (47%)	557 (47%)	1073 (47%)
High	589 (53%)	629 (53%)	1218 (53%) Missing: *n* = 349
Critical life events			
None	666 (52%)	617 (45%)	1283 (49%)
One or more	614 (48%)	743 (55%)	1357 (51%)
PHQ-9			
Below 10	1099 (93%)	1119 (88%)	2218 (90%)
10 or above	86 (7%)	155 (12%)	241 (10%)
Living with partner			
Yes	1120 (88%)	1134 (84%)	2254 (85%)
No	159 (12%)	224 (16%)	383 (15%) Missing: *n* = 3
Children in the household			
Yes	308 (24%)	250 (18%)	558 (21%)
No	970 (76%)	1108 (82%)	2078 (79%) Missing: *n* = 4

### Occupational and non-occupational domain

When dividing the working condition variables at the overall median, a marked difference between men and women appeared with respect to *job control*. While 62% of men (684) were in the higher control group, only 44% (518) of women were in the higher half of control values. There was no such difference in *appreciation by superior*, and high *quantitative demands* were also reported evenly by men and women.

*Family caregiving* responsibilities at home were reported by 74 persons (3%), caregiving responsibilities outside their own home by 236 persons (8.9%), such that 302 (11.5%) participants in all reported family care obligations. These numbers differed markedly by gender: 185 women (14%) reported care obligations, of which 34 provided care in their own home. 117 men (9%) reported care obligations, of which 40 provided care in their own home. 61% of total caregivers were female.

Mean *work hours* of employed persons were 35.3 h/week for caregivers and 36.4 h/week for non-caregivers. While a difference in the proportion of employees working part-time was clearly visible between employed men and women (5% vs. 48%), there was no such difference between caregivers and non-caregivers. The proportion of individuals working part-time was higher in caregivers (*n* = 78, 33%) than in non-caregivers (*n* = 461, 28%), reflecting the higher proportion of females in the caregiver sample.

*Critical life events* such as severe illness or divorce in the near past were reported by 48% of men (614) and by 55% of women (783). 63% of caregivers stated experiencing a critical life event in the near past as compared to 49% of non-caregivers.

### Depressive symptoms

The overall prevalence of *depressive symptoms* was approximately 10%. It differed strongly between men and women. While 86 men (7.3%) reported PHQ-9 values of ten or above, 155 women reported such high values (12.1%), with women having almost double the risk of depressive symptoms. Depressive symptoms were more common in the age group between 40 and 60 years of age, in which about 12% reported PHQ-9 values of 10 or above, and lower in the younger (36–40 years) and older (> 60 years) age groups, where the prevalence was about 5%.

Prevalence of depressive symptoms was higher in caregivers than in non-caregivers (13.5% vs. 8.5%). This did not simply reflect the higher number of women caregivers, because the difference was visible in both men (9% vs 7%) and women (15% vs. 12%).

**Fig. 1:**
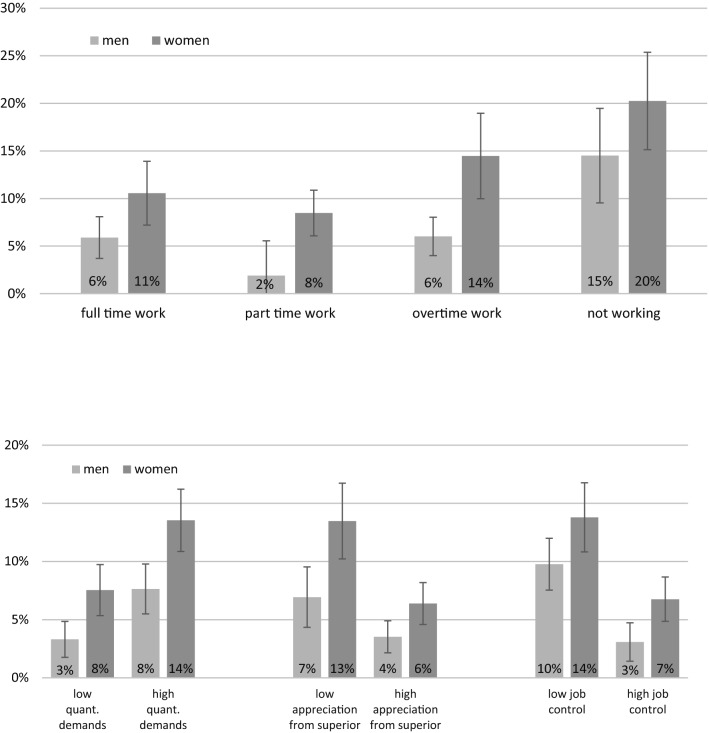
Prevalence of depressive symptoms in Wave 2 of the Study of Mental Health at Work 2, by gender and levels of working conditions. The prevalence is substantially higher in those who report low levels of appreciation by superior or influence at work vs. high levels, and in those who report high levels of quantitative work demands. It is highest in the non-working part of the sample

The proportion of individuals with depressive symptoms were roughly the same within groups working full-time or overtime, less in the group working part-time, both for men and for women. Prevalence was highest in the non-working part of the sample. The prevalence of depressive symptoms differed markedly between different levels of working conditions, especially for low vs. high levels of appreciation by superior as well as for low vs. high job control, as illustrated in Fig. 1.

### Regression analyses

The logistic regression model with interaction terms for family care obligations showed no significant interactions of family care obligations with any of the occupational variables, as presented in Table 3. In the stratified model, people with family care obligations had slightly higher odds ratios for all occupational variables. The highest odds ratios were seen for low appreciation from superior.

**Table 3:** Care-obligations stratified regression model

	Without family care obligations, *n* = 1883				With family care obligations, *n* = 237				interaction *p* value
	OR	95% CI		*p*	OR	95% CI		*p*	
Part time work	0.8	0.5	1.3	n.s	1.5	0.5	5.1	n.s	0.333
Overtime work	1.2	0.8	1.9	n.s	1.9	0.6	6.1	n.s	0.665
Low job control	2.0	1.3	2.9	0.001	2.4	0.9	6.8	n.s	0.913
Low appreciation from superior	2.7	1.9	4.0	< 0.001	2.8	1.1	6.8	< 0.05	0.780
High quantitative demands	1.5	1.0	2.2	< 0.05	2.6	0.9	7.3	n.s	0.338

Logistic interaction model for prevalence of depressive symptoms, adjusted for age, gender, CLE, partner, children

In the gender stratified analyses the associations in several of the independent variables indicated differences, as illustrated in Table 4. However, none of the interactions of occupational or non-occupational variables with gender were statistically significant. Most prominently, the associations of low job control and appreciation by superior with risk of depressive symptoms seemed to differ considerably between men and women. While the odds ratio for depressive symptoms was 1.6 for women with low job control vs. women with high job control, the odds ratio was 2.9 for men, i.e. the odds of having depressive symptoms were 2.9 times higher for men with low job control as compared to men with high job control. For women, low appreciation by superior had an odds ratio of 3.2, even larger than the odds ratio of job control, while for men the odds ratio for depressive symptoms associated with low appreciation was 2.1. While part time work had an odds ratio of 1 for women, it was associated with a lower prevalence of depressive symptoms in men, with an odds ratio of 0.4. Critical life events were strongly associated with risk of depression in both men and women, the association being stronger in men.

**Table 4:** Gender-stratified regression model

	men, *n* = 1020				women, *n* = 1101				Interaction *p* value
	OR	95% CI		*p*	OR	95% CI		*p*	
Part time work	0.4	0.0	3.0	n.s	1.0	0.6	1.7	n.s	0.368
Overtime work	1.1	0.6	1.9	n.s	1.4	0.8	2.5	n.s	0.488
Low job control	2.9	1.6	5.2	< 0.001	1.6	1.0	2.5	n.s	0.104
Low appreciation from superior	2.1	1.2	3.7	< 0.05	3.2	2.1	4.8	< 0.00*	0.227
High quantitative demands	1.9	1.0	3.7	< 0.05	1.5	1.0	2.3	n.s	0.542
CLE	3.0	1.6	5.4	< 0.001	2.3	1.5	3.7	< 0.001	0.597
Family care obligations	1.3	0.5	3.0	n.s	1.2	0.7	2.2	n.s	0.966
No partner	1.8	0.9	3.8	n.s	1.8	1.1	3.0	< 0.05	0.859
Children	0.8	0.4	1.6	n.s	0.9	0.5	1.7	n.s	0.866

Logistic interaction model and gender stratified model for prevalence of depressive symptoms, adjusted for age

### Sensitivity analyses

We repeated the regression analysis with the unimputed dataset, which did not substantially change any odds ratios. We also repeated the regression analyses using continuous variables for the occupational burden scales and work hours. No interaction was significant with family care obligations. The interaction of gender with job control was significant in the analysis of continuous variables, which had not been so in the dichotomous variable analyses.

## Discussion

The present study investigated the association of both occupational and non-occupational factors with the risk of depressive symptoms in German employees. To our knowledge, this is the first study to integratively examine these associations in a large representative German sample. We report three key results. First, consistent with previous research, job control is strongly associated with risk of depression. Second, low appreciation by superior is strongly associated with risk of depression. Third, the associations work factors with depressive symptoms seem to differ between caregivers and non-caregivers, and between men and women, though not strongly enough to show a significant statistical interaction. For women, appreciation by superior is the work factor with the strongest association of risk of depressive symptoms.

### Job control is strongly associated with risk of depressive symptoms

Work stress is an established influential factor for mental health problems. The Job Demand Control theory (Karasek and Theorell 1992) assumes that simultaneous exposure to high demands and low control causes emotional and/or physiological chronic stress. This stress reaction leads to deteriorated mental and physiological health. Job control is generally the more important factor of the two and has been found to be a significant predictor for mental health outcomes. Theorell et al. (2015) showed that there is consistent and well-established evidence for the association of job control with depressive symptoms. Our results confirm this importance: job control is a work factor strongly associated with risk of depressive symptoms. However, quantitative work demands are also associated with depressive symptoms.

### Low appreciation is strongly linked to depressive symptoms

The concept of *appreciation* is frequently discussed in the context of the fundamental needs of employees. One recent study highlights the importance of social relations and climate at work (Rochus Mummert 2016). While after 1 year on the job three out of four employees were satisfied with their work, this number was well over 90% in companies with an appreciative culture. However, while appreciation has been trained for many years as an essential component of “health-promoting leadership” to enable better productivity and to promote wellbeing, there is limited reliable scientific data on the relationship between appreciation by superior and mental health. Relational factors in general and their associations with (mental) health have not received much attention in psychosocial occupational epidemiology (Burr and d’Errico 2018). Though the original demand control model (Karasek 1979) was later extended to include the dimension of social support (Johnson and Hall 1988) to emphasize the relational aspect, it focuses on work task support and problem solving only, rather than on appreciation. Also, only few studies have taken up this extension of the DC model. And though in the ERI model appreciation is included as one aspect, it is usually not considered separately. Rather it is merged with financial reward and status into the “reward” dimension, which is then compared to the “effort” dimension. Nevertheless, there is moderate evidence for related concepts such as supervisor support or conflicts with superiors to be associated with depressive symptoms (Theorell et al. 2015).

In our study, appreciation by superior is a factor strongly associated with risk of depression. The magnitude of the association is comparable with that of CLE, which is recognized as one of the most important risk factors for depressive symptoms. The strong link we see between appreciation by superior and prevalence of depressive symptoms suggests that appreciation deserves more attention in psychosocial occupational epidemiology. Appreciation is a fundamental need of employees, reflected by positive feedback, originating from respect and esteem, and expressed through interest, attention and amenability. However, appreciation is not easy to implement. A superior who wants to practice real appreciation must think beyond traditional reward systems and recognize the value of a person regardless of their achievements. The extent to which mental health can be promoted through appreciation provides a difficult, but potentially rewarding, approach for future intervention studies.

### The association between working conditions and depressive symptoms is stronger in caregivers

Balancing family care obligations and work can be challenging for employees; therefore, employees can be differently affected by their occupational burdens (Lee et al. 2001). Our study shows stronger associations with depressive symptoms for all included occupational variables. None of these differences are statistically significant, however, since these interactions would need even larger samples to reach adequate statistical power. The largest difference in associations with depressive symptoms was visible in quantitative demands, where the odds ratio for caregivers was much higher than that for non-caregivers. Surprisingly, our study shows an odds ratio larger than one in caregivers for part time work, indicating that in caregivers working part time is associated with higher odds of depressive symptoms than working full time, while in non-caregivers, working part time is associated with a lower risk of depressive symptoms. However, this may also mean that in caregivers depressive symptoms are associated with higher odds of part time work, which cannot be distinguished in this cross-sectional study.

### The association between working conditions and depressive symptoms differs between men and women

As expected, women in our study had higher rates of depressive symptoms. This phenomenon has long been established, and while it differs in magnitude between countries, the difference persists even across cultures (Andrade et al. 2003). Depressive symptoms in our study were strongly associated with both appreciation and job control. However, the strength of these associations may differ between men and women. While appreciation by supervisor seems to be more important for women, job control appears to play a greater role for men. Similar results for job control have been presented in (Clumeck et al. 2009), who report stronger associations with depression related sick leave for men with lower job control.

Gender-differential associations of psychosocial work factors with stress have been reported before. For example, Vermeulen and Mustard (2000) found effects of job strain and social support to differ between men and women. Padkapayeva et al. (2018) also found differences in the effects of job control, job insecurity, and supervisor support on distress. Since women’s self-esteem is more dependent on external feedback, relational work characteristics are especially important for women. Therefore, the superior’s appreciation could possibly carry more weight, and a lack of appreciation could be a risk factor for the development of depressive symptoms. For men, low job control is linked with lower and conflicting with the cultural ideals of masculinity emphasizing power and dominance (Pudrovska and Karraker 2014). Low job control may, therefore, contribute to lower self-esteem and risk of depressive symptoms in men.

### Strengths and weaknesses

The SMGA study was an excellent basis for assessing the associations of occupational and non-occupational burdens with risk of depression in employees, because it comprises data from a very large representative sample of German employees. The study included data on working conditions as well as non-occupational burdens. When assessing mental health, interview-related and diagnostic biases were avoided using questionnaires. The study describes associations in the population of employees only, and only those who reported about their working conditions. Thus, associations may not be comparable to representative population studies because of the selected set of subjects. Even descriptive characteristics may not be population representative. For example, the proportion of women among caregivers in our study is lower than in Meyer 2006, which used German population representative data. Our sample does not contain the large part of the caring population that is retired, the majority of which are women.

Also, as our analyses concern the second wave of the study, the sample may have become biased by drop-out. Non-responder analyses have been conducted to look for differences between waves (d’Errico et al. 2021). Its results showed that men and women equally often continued to the second study wave, but that younger as well as unskilled employees did not continue to the second wave as often as older persons, managers and professionals did. Also, employees with lower workload and lower possibilities for development did not continue to the second wave as often as employees with a higher workload and higher possibilities for development. However, though these differences may cause some bias in the estimation of occupational burden prevalences, point estimates of associations are not affected, only their variance.

The study, however, highlights the situation of employed men and women, which is not captured in population representative studies.

The prevalence of depressive symptoms was highest in the non-working subsample. While this finding could be indicative of the adverse effects of unemployment and subsequent stressors like financial stress on mental health (Kessler 1997), it could also indicate the effect of depression on the ability to work. Indeed, in the association of work and private factors with depressive symptoms, reverse causation may be an issue, such that we cannot be certain to be seeing effects of work on risk of depressive symptoms, but may instead be seeing effects of depressive symptoms on work. Consequently, in this cross-sectional study we cannot make statements about the direction or the presence of causation.

Family care obligations are an established risk factor for increased levels of depression, stress, and mental problems. Caregivers are most stressed by the loss of autonomy over their time, which is determined by care obligations (Meyer 2006). We included family care obligations into the analysis, expecting associations with occurrence of depressive symptoms. However, these associations were not significant. Sensitivity analyses with continuous variables also dismissed family care obligations as insignificant. Only a null model including no covariates showed significant association of family care obligations with risk of depression.

Mean imputation for depressive symptoms was used. This causes a small decrease in variation and can lead to slightly lower p-values. Since the proportion of imputed valued was small (7%), and the SF-12 variables were very similar to the PHQ-9 questions, we opted for this uncomplicated method. Sensitivity analyses showed no substantial deviations from the unimputed model. For other scales, we did not impute any values. Reasons for missing values may include interview-related issues such as survey fatigue and social desirability, if participants do answer questions. In addition, missing values can be structural, if, for example, a person does not have a superior.

## Conclusion

In a representative sample of German employees, both the occupational and the non-occupational domain contain stressors significantly associated with risk of depression. Appreciation by superior and job control, together with critical life events, were the most important factors related to depressive symptoms. Surprisingly, appreciation, which has been neglected in psychosocial occupational research so far, was the most important factor in the entire sample. The gender differences with respect to appreciation and job control suggest that gender-sensitive prevention strategies should be developed and evaluated. The stronger associations for caregivers suggest that prevention may even more important for them. Furthermore, appreciation by superior needs more attention as an important psychosocial factor potentially influencing employee’s mental health. Future intervention studies must show whether the mental health of employees can be improved by training managers to promote a culture of appreciation.

## Data Availability

Not applicable.
